# A Method of Adding Binder by High-Pressure Spraying to Improve the Biomass Densification

**DOI:** 10.3390/polym12102374

**Published:** 2020-10-15

**Authors:** Xiaonan Ju, Kexin Zhang, Zhongjia Chen, Jianbo Zhou

**Affiliations:** 1 School of Engineering, Beijing Forestry University, Beijing 100083, China; juxiaonan@bjfu.edu.cn (X.J.); Kexin_Zhang@bjfu.edu.cn (K.Z.); 2 Forestry New Technology Research Institute, Chinese Academy of Forestry, Beijing 100091, China; 3 Beijing Forestry Machinery Research Institute of State Forestry and Grassland Administration, Beijing 100029, China

**Keywords:** biomass, densification, binder, high-pressure spraying method, relaxation density, compressive strength

## Abstract

In order to cut down the usage amount of binder, mix it more evenly with the biomass raw materials and improve the quality of pellets in the densification process, this study explored the feasibility of promoting the densification of biomass by using a high-pressure spraying method to add liquid binder. In the study, a high-pressure sprayer was used to spray saturated brown sugar water into sawdust for densification tests. A three-factor orthogonal experiment was designed to analyze the physical characteristics of the pellets under different variables. Through analysis of range and multiple linear regression, the effect curve was drawn to analyze the impact of the high-pressure spraying method on densification. The results showed that under low compaction pressure of 14.9 MPa, the raw materials with adding 6% saturated brown sugar water can be densified into pellets, while the raw materials without binder cannot. Moreover, compared with the method of adding binder by stirring, the high-pressure spraying method obtained the pellets with fewer cracks on the surface and increased the relaxation density of pellets by 8.65%. Under high compaction pressure (75, 100, 124 and 149 MPa), the high-pressure spraying method has a significant effect on increasing the relaxation density, not only on the compressive strength.

## 1. Introduction

Biomass, as a clean and renewable energy source, can significantly reduce net carbon emissions compared to fossil fuels and has a very broad development prospect [1]. Due to great concerns about the inevitable depletion of fossil resources, researchers have recently focused on developing new materials from biorenewable and sustainable sources. Biorenewable materials are widely used as reinforcement in many applications [2]. With the disadvantages of low bulk density and irregular shape and size, the biomass raw materials should be densified into higher density biomass pellets before applications, which helps with storage, handling and transportation [3]. The transformation technologies that are conductive to consuming biomass can be divided into three major groups: direct combustion routes, thermochemical routes (such as the transformation of biomass using steam gasification) and biochemical routes [4]. Biomass pellets as an important substitution of fossil fuels [5,6,7], which is widely used in industrial power generation and household cooking, will bring the benefits in environmental-friendly and economic aspects.

Process variables (such as compaction pressure) and material variables (such as particle size and binder) play an important role in achieving the good physical characteristics of biomass pellets such as durability, density and compressive strength. The compaction pressure is a very important variable during the densification process of biomass raw materials. The purpose is to destroy the original phase structure of the material and form a new phase structure to strengthen the cohesion between molecules, which makes the material become dense and enhances the strength of the pellets [8]. Razuan et al. found that pellets formed at higher pressures were found to be stronger and more uniform in shape over time [9]. The experiment conducted by Li Meihua [10] and Xing Lei [11] showed that increasing compaction pressure significantly increased the pellet density under the low pressure, and there is a maximum pressure beyond which no significant gain in the density of the pellets can be achieved.

In addition, the particle size of raw materials has a great impact on the densification effect of biomass at normal temperature and high compaction pressure [12]. Zhang Yan et al. reported that the density of pellets is inversely proportional to the particle size of raw materials [13]. P.d.grover et al. considered that sawdust particles should be no more than 6 mm [14], because the greater surface area of contact for the smaller particles during densification can produce stronger molecular attraction between particles to increase the relaxation density [15]. However, if the particle size of the raw material sample is too small, it is prone to moisture absorption. It is easy to crack or even shatter after desification, which restricts the densification effect of the pellets [16]. Therefore, adding binder with microparticles to biomass raw materials makes it adhere to the surface of biomass raw material, which can improve the quality of pellets to avoid the poor densification effect such as cracking on the surface or breaking.

At present, considering the power consumption in the biomass densification process, the energy consumption can be reduced by adding binder during densification. Xia Xianfei et al. indicated the biomass pellets with high compressive strength, high relaxation density and good durability can be obtained by the means of adding binder [17]. Ali Abedi et al. showed that the influence of binder on the porosity of biomass pellets is similar to that of compactness and mechanical strength [18]. Rumpf [19] observed that the binder, which plays an important role in production, transportation and storage [20], can improve the mechanical strength of pellets by forming the solid bridges between particles.

There are many kinds of binders, some of which can promote the densification of biomass at normal temperature and improve the quality of pellets. Tabil et al. found that the limestone as a binder in the alfalfa pelleting process can significantly improve the performance of biomass pellets [21]. Zhang Jun analyzed that urea-formaldehyde resin as the binder can promote the densification of bark at normal temperature [22]. Pfost studied the significant effect of lignin binders and bentonite on straw granulation [23], which can improve the hardness and durability of pelletized biomass.

Monosaccharide composition can be a binder which is helpful to enhance the hardness and stability of pellets [24]. The viscosity of sugar is caused by molasses. Brown sugar contains about 10% of molasses. Therefore, molasses, as a binder, is a byproduct of sugarcane production. It can be used as a binder for biomass pellets to improve the mechanical properties and fuel characteristics of pellets [25]. At present, domestic and foreign scholars have a wide range of research on sugar as a binder.

Mania et al. reported that the addition of chitosan to the filaments improved their ability to crystallize [26]. M.M. Manyuchi et al. found that molasses was essential for the production of high-quality pellets [27]. Singh et al. used syrup as a binder to help the densification of straw [28]. Kaliyan et al. found that molasses as a highly viscous binder adhered to the surface of biomass particles and acted as the solid bridge [29]. Majid Soleimani et al. showed that compared with other adhesives, molasses and fructose obtained better performance on the durability index of biomass pellets regardless of biomass type [30].

However, the former studies adopt the method of adding binder by stirring. This paper aims to testify a new method of binder addition using high pressure spraying. The purpose of this way is to spray the binder in the form of fine mist to cover sawdust particles evenly, so as to achieve the goal of mixing binder more uniformly with the biomass raw materials. Therefore, adding binder in this method not only can better atomize the liquid binder but also contributes to the densification of biomass.

According to the previous research, Kexin Zhang et al. [31] proved that adding brown sugar water by a spraying method is beneficial to the densification of biomass pellets, and has a great promotion effect on the improvement of biomass pellets’ physical characteristics. Furthermore, the optimal conditions for the densification of biomass pellets under the set factor level are obtained. In this paper, under the condition of known brown sugar water concentration (saturated brown sugar water) with the best densification effect, the influence of increased spraying pressure (10 MPa) on pellets quality was further explored, especially on the densification effect at low pressure compaction. This study used sawdust as the raw materials and saturated brown sugar water as a binder with the high-pressure spraying method, which was expected to achieve a better densification effect. A three-factor orthogonal experiment including the binder addition method, compaction pressure and binder addition ratio was designed, and the effects of variables on relaxation density and compressive strength of pellets were obtained. Based on a large number of data collected in the experiment, the relevant curves were drawn to further determine the impact of the high-pressure spraying method on the characteristic of pellets.

## 2. Materials and Methods

### 2.1. Materials

The raw material utilized in this research consisited of poplar sawdust (Heilongjiang province, China) (particle size: 1–5 mm). Considering that the physical and chemical properties of wood materials are greatly affected by temperature and humidity [32], the sawdust samples with no binder addition were sealed for storage after drying naturally. The stored material was dried at 105 °C for 6 h until the mass was constant and the moisture content was calculated. The moisture content of raw materials will increase after adding saturated brown sugar water. Meanwhile, for sawdust which densified under nomal temperature, the optimal moisture content is between 12% and 16% [33]. Considering that high moisture content might make pellets too soft and fracture easily, and excessive binders in industrial applications will lead to cost increasing, the proportion of binder in the variable should not exceed 10%. The research indicates that the compaction pressure acting on the pellets is between 111.2 and 194.6 MPa, whose density is between 0.95 and 1.3 g/cm^3^ (the relevant information shows that the density is about 1.1 g/cm^3^, which can meet the storage and transportation requirements), so no more pressure is needed during the densification to avoid energy waste [33]. Therefore, the compaction pressure values selected in this experiment are four gradient ranges of 75, 100, 124 and 149 MPa. The orthogonal test was designed, and three samples of each group were set for the repeated tests to take the average value. In the test, saturated brown sugar water was selected as a binder. Brown sugar made from pure sugarcane juice is granular, and saturated brown sugar water is made from brown sugar and water at the ratio of 2 [34].

### 2.2. Methods

#### 2.2.1. Moisture Content Determination

Referring to a method for determination of the moisture content of wood GB/T 1931-2009, the Equation (1) for calculating the moisture content is as follows:

(1) $$W=\;\frac{m_{1}-m_{0}}{m_{0}}\times 100\%$$

where: m_1_—the mass of wet sawdust (g); m_0_—the mass of absolute dry sawdust (g); W—the moisture content of sawdust (%).

The moisture content of the sawdust was measured by a rapid moisture meter and adjusted to 10% by adding water.

#### 2.2.2. Experimental Equipment

**Figure 1:**
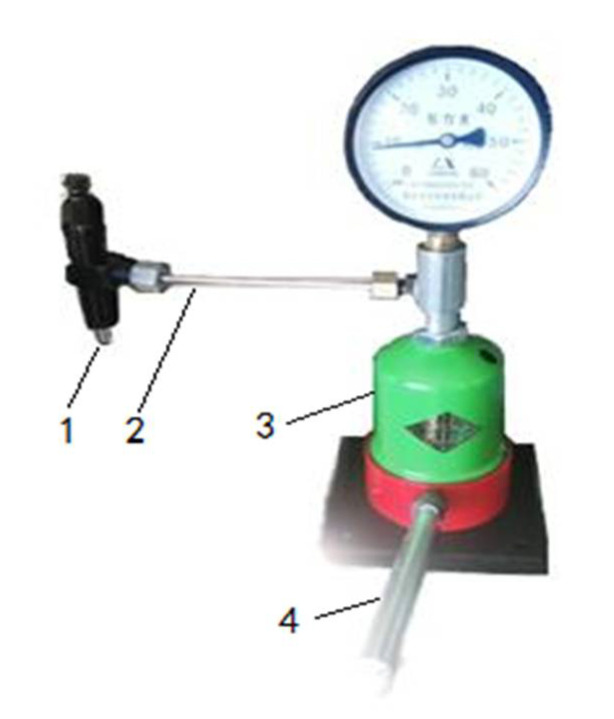
The high-pressure spraying device: 1—sprayer; 2—tubing, 3—hand pump; 4—compressive bar.

**Figure 2:**
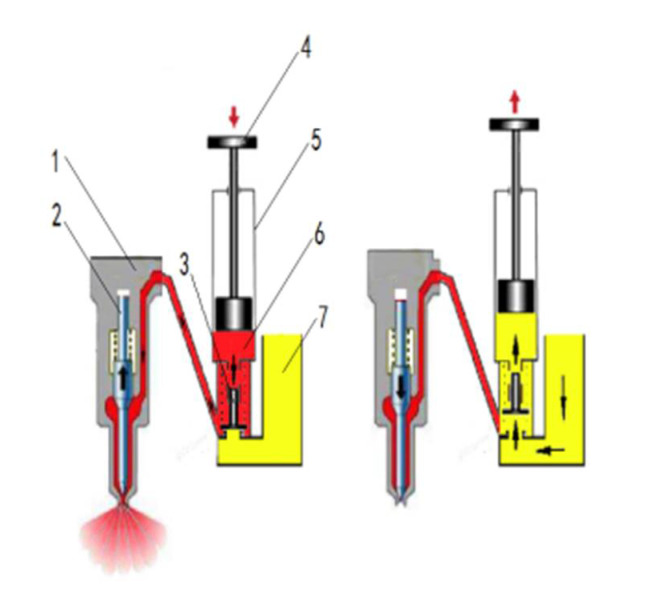
The schematic diagram of the spraying method: 1—sprayer shell; 2—needle valve; 3—one-way valve; 4—piston; 5—pump casing; 6—high-pressure liquid; 7—low-pressure liquid.

**Figure 3:**
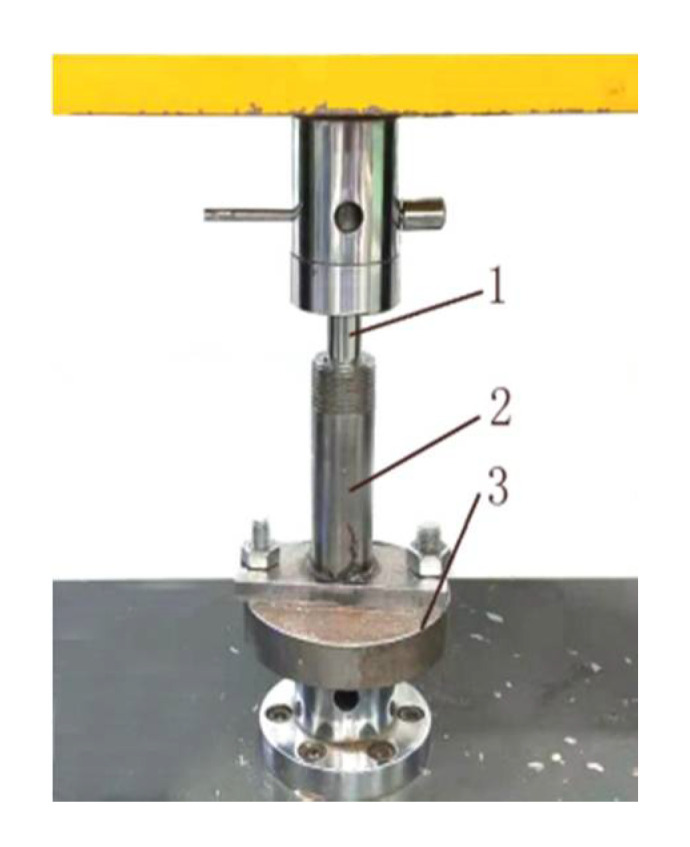
The diagram of compaction apparatus: 1—plunger; 2—die sleeve; 3—pedestal.

1. A high-pressure spraying device (model: XZ-PJ602L13, Shanghai, China)(pressure gauge, exit pressure: 10 MPa). In this test, compared with the 4 MPa sprayer in the previous study, the 10 MPa high-pressure sprayer (Figure 1) was used for spraying. It consists of a sprayer (1) (nozzle diameter: 0.6 mm), and a hand pressure pump (3) (height: 33 cm), which are connected by tubing (2). Under normal conditions (Figure 2), the needle valve (2) is closed under the spring pressure. When the piston (4) is pressed, liquid under the piston (4) is pressurized. The high -pressure liquid (6) enters the sprayer through the one-way valve and tubing; then, the needle valve (2) overcomes the spring pressure and rises under the push of the high-pressure liquid (6). Afterwards, the binder is sprayed from the nozzle hole at the tip of the needle valve (2). Under high pressure, the liquid binder will spray out as a mist. The schematic diagram is shown in Figure 2.
2. Plunger and die (inner diameter: 16 mm; length: 125 mm). This test used following compaction apparatus for densification (Figure 3). During densification, the plunger exerted pressure on the raw materials in the die to densify the biomass raw materials.
3. Microcomputer-controlled electronic universal testing machine (Model: 4050, Reger, Shenzhen, China), electronic balance(Model: SF-400A, Shanghai, China) (accuracy: 0.01 g), moisture tester (model: SC69-02, Shanghai, China), vernier caliper(precision: 0.01 mm), etc.

#### 2.2.3. Pretreatment of Sawdust and Binder

In the production of biomass pellets, sawdust particles were mixed with binder particles according to the experimental design. Two methods including stirring and high-pressure spraying were, respectively, employed to mix the biomass raw materials with binder. High-pressure spraying method: 20 g poplar sawdust including three samples was evenly spread in a 20 cm × 20 cm square plate, and the plate was placed on the electronic scale to weigh. It was calculated that 3%, 6% and 9% of the samples, respectively, corresponded to 0.62, 1.28 and 1.98 g brown sugar water. The distance between the sprayer and the sawdust was 90 cm, and high-pressure spraying was performed under the outlet pressure of 10 MPa. Then, the mixed materials were put into the sealed bag and placed for 10 min before the densification. Stirring method: the saturated brown sugar water was sprinkled on the surface of poplar sawdust with a needle; then, stirring for 2 min made it mix with biomass raw materials.

The 7 g modulated mixture was filled into the die. Then the universal testing machine was used to compact the biomass materials in the die at the speed of 5 mm/min. After reaching the compaction pressure value set in the test, the plunger stayed in this position for 30 s (retention time), and then exited from the die. A change in the temperature and relative humidity leads to higher moisture absorption and lower mechanical strength [35]. Because the relative humidity of the working environment was about 80%, the densified biomass pellets were put into a sealed bag after being taken out from the die to avoid moisture absorption.

#### 2.2.4. Pretest—the Effect of High-pressure Spraying Method at Low Pressure Compaction

1. Test method. At a pressure of 14.9 MPa, adding 6% binder was taken as an example. The densification test was carried out on three groups of samples including no binder addition, 6% brown sugar water addition by stirring and 6% brown sugar water addition by highly-pressured spraying. Each set of tests was repeated 3 times, and the average value was calculated to compare the densification effect. The pretest aims to verify the feasibility of the high-pressure spraying method: that is, whether this method promotes the densification of biomass.
2. Evaluation index.
  - Densification effect. At the same compaction pressure, the experiments under different densification conditions were compared. The evaluation criteria were whether the sawdust can be densified into pellets and the surface quality of the pellets after densification.
  - Relaxation density.

After the pellet was removed from the die, due to elastic deformation and stress relaxation, its compression density gradually decreased, and the density tended to stabilize after a certain time. At this time, the density of the pellet is also called relaxation density. The higher the density, the higher the energy or volume ratio. Therefore, from the perspective of transportation, storage and carrying, high-density products are preferred [36]. The length and diameter of the pellets with a relaxation time of 2 h were measured with an electronic vernier caliper. The length of three densified pellets per group was measured, and the average value was calculated. In order to ensure the accuracy of measurement, the diameter of the pellet was measured from three positions including the front, middle and back end, and the average value was taken. The mass of the biomass pellets was measured by electronic balance (0.01 g). The Equation (2) for calculating the relaxation density is as follows:

(2) $$\rho \;=\frac{4m}{{\;\pi d}^{2}h}$$

where: ρ—relaxation density (g/cm^3^); m—the mass of a biomass pellet (g); d—the diameter of biomass pellet (cm); h—the length of biomass pellet (cm).

#### 2.2.5. The Effect of the High-Pressure Spraying Method at High Pressure Compaction

1. The table L16 (2 × 4^2^) was used to design a three-factor mixed level orthogonal test (Table 1).

2. Evaluation index:
  - Relaxation density.
  - Compressive strength.

As compressive strength is directly related to the transportation and storage of biomass pellets, it is widely used as an important index to evaluate the quality of pellets [37]. The length of three densified pellets per group was measured, and the average value was taken. In order to ensure the accuracy of results, three samples were taken from each group for each test, and the average value was calculated. The biomass pellet’s axis was perpendicular to the horizontal plane of universal mechanical testing machine, and a small amount of pressure was applied to ensure that the sample was fixed. When the compressive strength test began, the compressive bar moved downward at a speed of 5 mm/min until the sample cracked completely [38]. The compressive strength of the pellet was calculated from the maximum load and the dimensions of the pellet. The calculation equation of compressive strength Equation (3) is as follows:

(3) $$\sigma_{t}=4\;\frac{F_{\max}}{{\pi D}_{t}{}^{2}}$$

where: $\sigma_{t}$—compressive strength (Pa); $F_{\max}$—maximum axial force (N); $D_{t}$—the diameter of biomass pellet (m).

In order to accurately measure the value of the maximum axial force, this study took the highest point of the failure curve drawn by the universal mechanical testing machine as the failure point, and the corresponding axial force is the maximum axial force.

#### 2.2.6. Statistical Software

In this paper, statistical analysis of the results was carried out using the IBM SPSS Statistics 24 (IBM, Armonk, NY, USA) based on a three-factor mixed level orthogonal test. With complete data input, editing, statistical analysis, reports, graphics production and other functions, SPSS was used for regression analysis of experimental data.

## 3. Results and Discussion

### 3.1. Densification Experiment under Low Compaction Pressure

**Figure 4:**
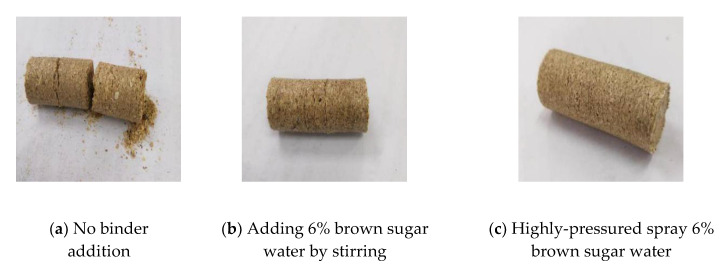
Densification effect.

In this study, the preliminary experiment was conducted to densify the sawdust with different binder addition methods under the pressure of 14.9 MPa. The densification effect is shown in Figure 4, and the experimental results are shown in Table 2.

a, b and c, respectively, represent no binder addition, adding 6% brown sugar water by stirring and highly-pressured spray 6% brown sugar water.

It can be seen from the Table 2 and Figure 4 that under a low compaction pressure, which is 14.9 MPa, the biomass raw materials without saturated brown sugar water binder cannot be compacted, and cracks on the surface of pellets and dropping fragment occur easily, which promotes pellet fracture. Biomass raw materials can be densified in both stirring and high-pressure spraying methods, but compared with the method of adding binder by stirring, the better densification effect with fewer cracks can be obtained by a high-pressure spraying method.

Table 2 shows that the relaxation density of the pellets obtained by using high pressure to spray brown sugar water increased by 8.65% compared with the method of adding brown sugar water by stirring. Therefore, under the low pressure, the high-pressure spraying method significantly increases the relaxation density of the biomass pellets and is helpful to the densification of biomass.

### 3.2. Densification Experiment under High Compaction Pressure

#### 3.2.1. Experimental Results of Relaxation Density

Table L16 (2 × 4^2^) was used to design a three-factor mixed level orthogonal test (Table 3). There were 16 sets of tests, each set of tests was repeated 3 times, and the average value was calculated. A, B and C, respectively, represent compaction pressure, binder addition ratio and binder addition method. Y1and Y2, respectively, represent the relaxation density (g/cm^3^) and compressive strength (MPa).

#### 3.2.2. Range Analysis

Range analysis table (Table 4) was obtained by analyzing the range R of biomass pellets’ relaxation density based on Table 3. K_i_ represents the sum of the corresponding experimental results when the level sign on any column is i, and k_i_ represents the average value of K.

**Figure 5:**
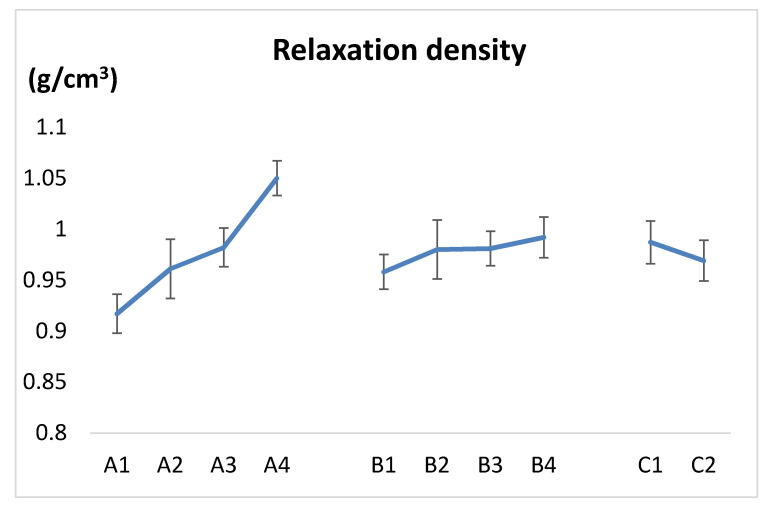
Factors and levels—relaxation density; A—compaction pressure (A1–A4: 75, 100, 124 and 149MPa); B—binder addition ratio (B1–B4: 0%, 3%, 6% and 9%); C—binder addition method(C1–C2: spraying and stirring).

According to the range analysis, the larger the range value, the greater the influence of this factor on this index, and this factor is counted to be the main factor on the quality of biomass pellets. It can be seen from Table 4 that the influence degree of each factor on the relaxation density of pellets is as follows: compaction pressure > binder addition ratio > binder addition method. The corresponding effect curve is shown in Figure 5. With the increase in compaction pressure and binder addition ratio, the relaxation density of pellets increases. The effect of the high-pressure spraying method on the relaxation density of pellets is greater than that of stirring. According to the k value, the optimal combination of experimental factors can be determined: that is, A4B4C1.

#### 3.2.3. Multiple Linear Regression Analysis

The regression coefficient test table (Table 5) was obtained by using SPSS software and analyzing linear regression based on Table 3.

According to the standardized coefficient and t test, the significance of the test variables was evaluated as follows:

n = 16, k = 3; regression coefficient test degree of freedom = n − k − 1 = 16 − 3 − 1 = 12. Looking at the table, when alpha = 0.05, t (12) = 2.179.

The standardized coefficient of pressure is 0.922, close to 1, and the t value is 11.993, which is much higher than the critical value t, indicating that pressure has a significant influence, and pressure is the most significant factor.

The standardized coefficient of the binder addition ratio is 0.226, and the t value is 2.940, which is greater than the critical value t, indicating that the binder addition ratio has a significant influence. The binder addition ratio is a significant factor, but the significance is less than the pressure.

The standardized coefficient of the binder addition method is −0.168, and the absolute value of t is 2.180, which is greater than the critical value t, indicating that the binder addition method has a significant influence. The binder addition method is a significant factor, but the significance is less than the pressure and the binder addition ratio.

In conclusion, beta1, beta2 and beta3 all passed the test. This indicates that the influence of compaction pressure and binder addition ratio on the relaxation density of biomass pellets is significant. Although the binder addition method has less influence than the compaction pressure and binder addition ratio, it also has a remarkable effect on the relaxation density.

### 3.3. Destruction Test

#### 3.3.1. Experimental Results of Compressive Strength

The experimental results of the compressive strength of biomass pellets are shown in Table 6.

#### 3.3.2. Range Analysis

Range analysis table (Table 7) was obtained by analyzing the range R of biomass pellets’ relaxation density based on Table 6. K_i_ represents the sum of the corresponding experimental results when the level sign on any column is i, and k_i_ represents the average value of K.

**Figure 6:**
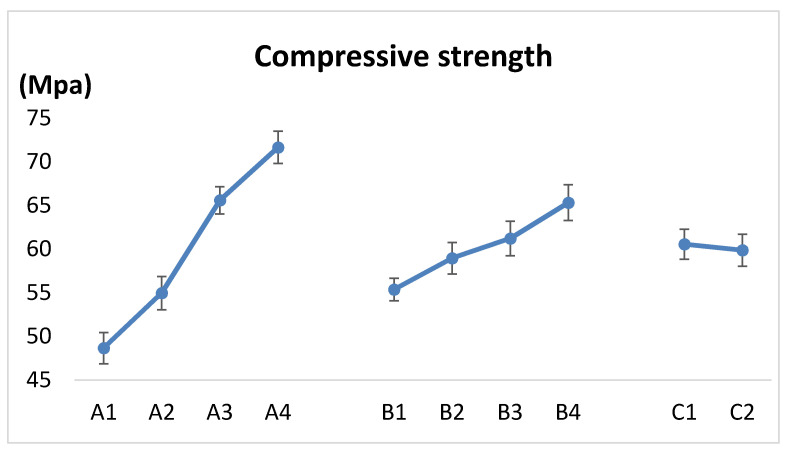
Factors and levels—compressive strength: A—compaction pressure (A1–A4: 75, 100, 124 and149 MPa); B—binder addition ratio (B1–B4: 0%, 3%, 6% and 9%); C—binder addition method(C1–C2: spraying and stirring).

It can be seen from Table 7 that the influence on the degree of each factor on relaxation density of pellets is as follows: compaction pressure > binder addition ratio > binder addition method. The effect curve is shown in Figure 6. With the increase in compaction pressure and binder addition ratio, the compressive strength of pellets increases. The effect of the high-pressure spraying method on the compressive strength of pellets is greater than that of stirring. According to the k value, the optimal combination of experimental factors can be determined: that is, A4B4C1.

The pellets have a certain compressive strength after densification, and the compressive strength of the pellets obtained by the high-pressure spraying method is higher. The test results show that the high-pressure spraying method assisted in increasing the strength of the pellets, and the compressive strength of the pellet can reach more than 45 MPa when it is axially compressed.

#### 3.3.3. Multiple Linear Regression Analysis

The regression coefficient test table (Table 8) was obtained by using SPSS software and analyzing linear regression based on Table 6.

According to the standardized coefficient and t test, the significance of the test variables was evaluated as follows:

n = 16, k = 3; regression coefficient test degree of freedom = n – k − 1 = 16 – 3 − 1 = 12. Looking at the table, when alpha = 0.05, t (12) = 2.179.

The standardized coefficient of pressure is 0.909, close to 1, and the t value is 16.322, which is much higher than the critical value t, indicating that pressure has a significant influence, and pressure is the most significant factor.

The standardized coefficient of the binder addition ratio is 0.367, and the t value is 6.594, which is greater than the critical value t, indicating that the binder addition ratio has a significant influence. The binder addition ratio is a significant factor, but the significance is less than the pressure.

The standardized coefficient of the binder addition method is −0.036, which is approximately equal to 0,and the absolute value of t is 0.64, which is less than the critical value t, indicating that the binder addition method has no significant influence. The binder addition method is not a significant factor.

In sum, only beta1 and beta2 passed the test, while beta3 failed the test. This shows that the influence of compaction pressure and binder addition ratio on the compressive strength of biomass pellets is significant, but the effect of the binder addition method is not.

## 4. Conclusions

Based on the similar type of research, this paper further explored the influence of increased spraying pressure (10MPa) on pellet quality, especially on the densification effect at low pressure compaction. This experiment used the method of spraying binder with a high-pressure to improve the quality of biomass pellets using poplar sawdust as a raw material and saturated brown sugar water as a binder. An orthogonal experiment was designed to test two physical properties of biomass pellets—which are compressive strength and relaxation density under different compaction pressure—different binder addition ratio and different binder addition methods, and then the range and multiple linear regression according to index data were analyzed. Based on experiments and data analysis, it is found that saturated brown sugar water as a binder improves the relaxation density of biomass pellets, and the densification force between sawdust particles tends to increase slowly. Under the low pressure, the high-pressure spraying method can make the sawdust and the binder mix more evenly, which causes the relaxation density of the pellets to increase by 8.65%. With the significant improvement of the densification effect, the high-pressure spraying method is helpful for the densification. Compared with the method of adding binder by stirring under high pressure, the relaxation density and compressive strength of the pellets densified by the high-pressure spraying method increased slightly, particularly for the significant impact on the improvement of pellets’ relaxation density.

## Figures and Tables

**Table 1:** Orthogonal test table of three factors mixed level.

Level	Factor		
	Compaction Pressure (MPa)	Binder Addition Ratio (%)	Binder Addition Method
1	75	0	Spraying
2	100	3	Stirring
3	124	6	
4	149	9	

**Table 2:** Test results of relaxation density and surface quality of pellets.

Factor	Relaxation Density (g/cm³)	Surface Quality of Pellets
**a**	0.588 ± 0.004	Many large cracks on the surface and fracture easily
**b**	0.624 ± 0.005	Less large cracks and not easy to fracture
**c**	0.678 ± 0.002	Tiny cracks on the surface

**Table 3:** Relaxation density test results of pellets. L16 (2 × 4^2^).

Test group	A (MPa)	B (%)	C	Y_1_ (g/cm³)
1	75 (1)	0 (1)	Spraying (1)	0.897 ± 0.010
2	75 (1)	3 (2)	Stirring (2)	0.903 ± 0.005
3	75 (1)	6 (3)	Spraying (1)	0.933 ± 0.027
4	75 (1)	9 (4)	Stirring (2)	0.936 ± 0.028
5	100 (2)	0 (1)	Stirring (2)	0.924 ± 0.029
6	100 (2)	3 (2)	Spraying (1)	0.979 ± 0.042
7	100 (2)	6 (3)	Stirring (2)	0.948 ± 0.005
8	100 (2)	9 (4)	Spraying (1)	0.993 ± 0.038
9	124 (3)	0 (1)	Spraying (1)	0.976 ± 0.007
10	124 (3)	3 (2)	Stirring (2)	0.982 ± 0.042
11	124 (3)	6 (3)	Spraying (1)	0.995 ± 0.016
12	124 (3)	9 (4)	Stirring (2)	0.977 ± 0.010
13	149 (4)	0 (1)	Stirring (2)	1.035 ± 0.021
14	149 (4)	3 (2)	Spraying (1)	1.056 ± 0.025
15	149 (4)	6 (3)	Stirring (2)	1.049 ± 0.018
16	149 (4)	9 (4)	Spraying (1)	1.062 ± 0.002

**Table 4:** Range analysis table.

		A	B	C
Y_1_	K1	3.669 ± 0.070	3.832 ± 0.067	7.892 ± 0.168
	K2	3.844 ± 0.114	3.920 ± 0.114	7.752 ± 0.158
	K3	3.928 ± 0.075	3.924 ± 0.066	—
	K4	4.202 ± 0.066	3.968 ± 0.078	—
	k1	0.917 ± 0.019	0.958 ± 0.017	0.987 ± 0.021
	k2	0.961 ± 0.029	0.980 ± 0.029	0.969 ± 0.020
	k3	0.982 ± 0.019	0.981 ± 0.017	—
	k4	1.050 ± 0.017	0.992 ± 0.020	—
	R	0.133 ± 0.036	0.034 ± 0.036	0.018 ± 0.041
	Optimal level	A4	B4	C1

**Table 5:** Regression coefficient test.

Coefficient ^a^					
Model		Unnormalized Coefficient	Normalized Coefficient	t	Significance
		Standard Error	Beta		
1	(constant)	0.021		38.424	0.000
	A(MPa)	0.001	0.922	11.993	0.000
	B(%)	0.001	0.226	2.940	0.012
	C	0.008	−0.168	−2.180	0.050

^a^ The dependent variable: relaxation density(g/cm^3^).

**Table 6:** Compressive strength test results of pellets.

Test Group	A(MPa)	B(%)	C	Maximum Axial Force (KN)	Y2 (MPa)
1	75 (1)	0 (1)	Spraying (1)	9.10 ± 0.16	45.26 ± 0.78
2	75 (1)	3 (2)	Stirring (2)	9.37 ± 0.45	46.60 ± 2.24
3	75 (1)	6 (3)	Spraying (1)	9.64 ± 0.44	47.95 ± 2.19
4	75 (1)	9 (4)	Stirring (2)	11.03 ± 0.39	54.86 ± 1.94
5	100 (2)	0 (1)	Stirring (2)	9.65 ± 0.28	47.99 ± 1.39
6	100 (2)	3 (2)	Spraying (1)	11.36 ± 0.44	56.50 ± 2.16
7	100 (2)	6 (3)	Stirring (2)	11.38 ± 0.33	56.60 ± 1.63
8	100 (2)	9 (4)	Spraying (1)	11.82 ± 0.49	58.79 ± 2.43
9	124 (3)	0 (1)	Spraying (1)	12.43 ± 0.17	61.82 ± 0.86
10	124 (3)	3 (2)	Stirring (2)	13.02 ± 0.22	64.76 ± 1.10
11	124 (3)	6 (3)	Spraying (1)	13.51 ± 0.42	67.19 ± 2.08
12	124 (3)	9 (4)	Stirring (2)	13.80 ± 0.45	68.63 ± 2.24
13	149 (4)	0 (1)	Stirring (2)	13.35 ± 0.43	66.40 ± 2.14
14	149 (4)	3 (2)	Spraying (1)	13.68 ± 0.34	68.04 ± 1.71
15	149 (4)	6 (3)	Stirring (2)	14.71 ± 0.39	73.16 ± 1.96
16	149 (4)	9 (4)	Spraying (1)	15.89 ± 0.32	79.03 ± 1.58

**Table 7:** Range analysis table.

		A	B	C
**Y_2_**	K1	194.67 ± 7.15	221.47 ± 5.17	484.58 ± 13.79
	K2	219.88 ± 7.61	235.90 ± 7.21	479.00 ± 14.64
	K3	262.40 ± 6.28	244.90 ± 7.86	—
	K4	286.63 ± 7.39	261.31 ± 8.19	—
	k1	48.67 ± 1.79	55.37 ± 1.29	60.57 ± 1.72
	k2	54.97 ± 1.90	58.97 ± 1.80	59.87 ± 1.83
	k3	65.60 ± 1.57	61.22 ± 1.97	—
	k4	71.66 ± 1.85	65.33 ± 2.05	—
	R	22.99 ± 3.64	9.96 ± 3.34	0.7 ± 3.55
	Optimal level	A4	B4	C1

**Table 8:** Regression coefficient test.

Coefficient ^a^					
Model		Unnormalized Coefficient	Normalized Coefficient	t	Significance
		Standard Error	Beta		
1	(constant)	2.902		6.976	0.000
	C	0.020	0.909	16.322	0.000
	B(%)	0.162	0.367	6.594	0.002
	A(MPa)	1.089	−0.036	−0.640	0.534

^a^ The dependent variable: relaxation density(g/cm^3^).
